# Migration and Caesarean Section Birth in the United Kingdom: A Secondary Analysis of Born in Bradford Data

**DOI:** 10.1111/birt.70052

**Published:** 2026-02-04

**Authors:** Victoria Cadman, Rachael Spencer, Hora Soltani

**Affiliations:** ^1^ College of Health, Wellbeing and Life Sciences Sheffield Hallam University Sheffield UK

**Keywords:** caesarean birth, migrant, United Kingdom

## Abstract

**Background:**

Caesarean section (C‐section) rates in the United Kingdom continue to increase and are a concern. Births to migrants account for 30.3% of live births in England and Wales. Other international studies have observed varying rates of C‐section for migrant populations in comparison to women born within the country itself. Comparison of incidence rates of Caesarean section birth between migrant populations and women born in the United Kingdom (UK) was undertaken to inform the UK context and address an existing dearth of data.

**Methods:**

This study included analysis of 11,361 records from the Born in Bradford cohort study. Binomial logistic regression analysis was performed to estimate crude and adjusted odd ratios (aOR) with 95% confidence intervals (CI) for the incidence of total, elective, and emergency C‐section births between migrant populations and UK‐born women.

**Results:**

Women from “South Asia” and “Central Europe, Eastern Europe, and Central Asia” demonstrate lower incidences of total C‐section with a significantly lower elective C‐section. Women from Sub‐Saharan Africa demonstrate significantly high rates of total C‐section (38% increased odds).

**Discussion:**

High variation in the incidence of C‐section amongst migrant populations was observed, replicating findings from the few other international studies. Further in‐depth exploration is required to understand the impact of this variation on maternal and neonatal health disparities, and to assess the contribution of potential pathophysiological and sociocultural factors on related decision‐making processes.

## Graphical abstract

This secondary analysis of Born in Bradford data provides comparison of caesarean section birth rates (total, elective, and emergency) between migrant populations and UK‐born women. Findings show variations between migrant populations with some experiencing significantly lower, and others significantly higher incidence rates.

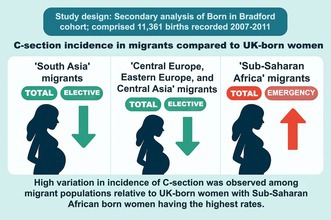

## 1 Introduction

Global migration continues to increase, with women representing nearly half of migrants [1]. In the United Kingdom (UK) data shows that 33.9% of all live births in England and Wales in 2024 were to migrants (defined as non‐UK born mothers), forming the highest proportion since records began and continuing the long‐term trend of an increasing overseas‐born population [2].

Global rates of Caesarean section (C‐section) have continued to increase without clear rationale [3] despite guidance from the World Health Organization (WHO) defining C‐section rates of above 10%–15% as “indicative of unnecessary and therefore unethical surgical intervention” [4]. Although with a medical indication, C‐section could be a life‐saving procedure, recent evidence demonstrates a higher risk of birth complications for women with a history of C‐section [5]. The UK has continued to report increasing numbers of C‐section with current figures at 41.1% for total C‐sections, consisting of 18.3% for electives and 23.1% for emergency C‐section [6]. Suggestions made for this trend include the changing complexity of women's health and sociodemographic circumstances [7].

Whilst C‐section is not solely attributed to births by migrant women, given their proportion of the birthing population (33.9%) and social and cultural complexities around migration and resettlement, along with prior perceptions of C‐section from their country of origin, exploration of this population group is warranted. Review studies have recognized variance in the incidence of C‐section between differing migrant populations globally, with some posing an increased likelihood and others a reduction [8, 9, 10]. However, little is known about the UK context, with only few studies examining either small specific migrant populations [11, 12, 13, 14, 15] or a large collective group of migrants [16]. The recent report by MBRRACE‐UK [17] continues to demonstrate the link between maternal morbidity, mortality, and poorer birth outcomes of women of Black, Asian, and Minority Ethnicities, with another study demonstrating a higher risk of postpartum hemorrhage across all ethnic minority groups, including when women underwent C‐section [18]. Whilst migrants do not exclusively form these groups of women, migrants are considered within these categories. It is equally important to identify populations indicating lower incidence rates from which to learn for informing interventions to enhance birth experience, reduce C‐section rates, or establish whether the lower rate is a consequence of missed opportunity for appropriate elective management resulting in poorer outcomes.

The objective of this study was to compare C‐section births of migrant populations to women born in the UK using a pre‐existing diverse dataset. This would subsequently allow identification of populations of interest for future work that could inform practice and lead to the improvement of birth experiences and related outcomes.

## 2 Methods

### 2.1 Setting and Context

This retrospective analysis is an extension of a previous project analyzing the intergenerational impact of migration on lifestyle behaviors, infant feeding, and pregnancy outcomes and utilized an existing data set from the Born in Bradford (BiB) family of studies [19]. BiB is an internationally recognized research programme which aims to examine factors that impact on health and development perinatally, during childhood and subsequent adult life, as well as those influencing their parent's health and wellbeing. Design of the cohort study involved wide consultation with community, neighborhood and faith groups and determined areas of public concern and resulted in strong support for the study with over 80% of eligible women consenting to take part in the study [20]. Bradford has a relatively high proportion of migrant communities, the most recent population estimates based on 2021/2022 census data detail this as 18.7% of the population, slightly above the average of 16.8% for England and Wales, with 45.2% of births to migrants which is higher than the 33.9% observed nationally [2, 21]. Census data closest to the BiB data collection was in 2011 and shows that Bradford had a migrant population of over 15% compared to the England and Wales average of 13% [22] with births to migrant mothers in 2010 reported as 25% [23]. This difference is reflective of the increase that has been seen nationally over this time period. This is likely to have resulted from changes in geopolitics, expanding war and conflicts, and changes in migration policy leading to increased rates of migration, along with higher total fertility rates amongst migrant populations due to their age profile and cultural and family orientations. The proportions of all births to women from different countries of origin are largely consistent with the distribution of country of origin for migrants residing in the UK. However, Bradford does demonstrate a higher proportion of migrants of Pakistani origin. The protocol for recruiting women for the data set utilized in this analysis is reported elsewhere [24].

### 2.2 Data Analysis

The data set comprised 11,396 birth records of women giving birth in Bradford between 2007 and 2010. For this analysis, records where the mother's country of birth was either missing, the mother had declined to answer, or where the country of birth was illegible from the free text response were excluded. Further records were excluded where there was missing data for a variable that would be used in further analysis with the exception of booking BMI. Records with missing booking BMI were included in the analysis as this would exclude those potentially experiencing factors resulting in a missing booking BMI that may disproportionately affect migrant women, such as not accessing maternity services and transfer of care, particularly if migration occurred during the pregnancy. After exclusions, 11,361 records were included in the analysis.

For the purpose of our study, the definition of migrant was “not born in the United Kingdom”. Women were grouped for analysis utilizing their stated country of birth into regional populations following Global Burden of Disease (GBD) super‐regions which groups countries based on epidemiological similarity as well as geographical closeness [25]; “High‐income”, “Central Europe, Eastern Europe, and Central Asia”, “North Africa and Middle East”, “Sub‐Saharan Africa”, “South Asia”, “Southeast Asia, East Asia, and Oceania” and “Latin America and Caribbean”. Table S1 provides details of the countries of origin of the women included in our analysis.

For determining elective (category 4) and emergency (categories 1–3) C‐sections, variables in the data set were reviewed as there were some discrepancies between the recorded onset of labor and other information recorded in the data. Therefore, for the purpose of our analysis, any C‐section which had an onset of labor recorded as “spontaneous” or any type of induction was deemed an emergency C‐section. C‐sections where any duration of labor time and/or rupture of membranes was recorded that had originally been scheduled as an elective were considered as emergencies. This was because women in this situation would at a minimum be considered as a category 3, emergency “non‐urgent” case requiring early delivery that would now be taking place prior to the chosen date of surgery and outside of scheduled elective provision [26]. All other C‐sections, those clearly identified as electives and those where there was no onset of labor unless the gestational age indicated an emergency, were considered as elective.

Statistical analysis was performed utilizing IBM‐SPSS Statistics (Version 26). Descriptive analysis was performed for demographic data with independent t–test used to compare means between each migrant population and the UK born women. Binomial logistic regression analysis was used to estimate crude and adjusted odds ratios with 95% confidence intervals (CI) for the incidence of C‐section versus vaginal birth for migrant population groups when compared to UK born women. ORs were then adjusted for the following: Model 1: maternal age and booking BMI; Model 2: maternal age, booking BMI, Index of Multiple Deprivation (IMD) quintile, diabetes (pre‐existing and gestational), hypertensive disorders (pre‐existing and pregnancy related but excluding those which occurred in labor only), smoking during pregnancy, parity, gestational age, and infant birth weight.

## 3 Results

Characteristics of the study population are provided in Tables 1 and 2. Considering the migrant population in comparison to UK born women, the mean maternal age was significantly older (*p* < 0.001), mean BMI was significantly lower (p < 0.001), migrant women were more likely to be married, less likely to be in employment or study, and were more likely to be in the most deprived quintiles of IMD. In terms of health and pregnancy, migrant women had a higher incidence of diabetes (12.0% compared to 6.3%), smoked less (2.9% of the population compared to 24.3% of UK born women), mean gestational age was found to be significantly lower (*p* < 0.05) as was the mean infant birthweight (*p* < 0.001). However, the breakdown of migrants into regional groups demonstrates variance across all variables between different regional migrant populations. For all migrant populations, the most common age of migration to the UK was during adulthood with this accounting for the majority of migrants in all but one regional group, ‘High‐income’ country migrants, who showed a higher proportion of women who had migrated whilst under the age of 12 years.

**TABLE 1:** Socio‐demographic characteristics of the study population.

Variable	Category	Total population *n* = 11,361	UK born women *n* = 7183 (63.2%)	Migrant women *n* = 4178 (36.8%)	South Asia *n* = 3284 (28.9%)	Central Europe, Eastern Europe, and Central Asia *n* = 288 (2.5%)	Sub‐Saharan Africa *n* = 241 (2.1%)	High‐income *n* = 128 (1.1%)	Southeast Asia, East Asia, and Oceania *n* = 115 (1.0%)	North Africa and Middle East *n* = 107 (0.9%)	Latin America and Caribbean *n* = 15 (0.1%)
Age at delivery (years)	Mean	27.58	27.11	28.39***	28.37**	26.20**	29.57***	27.89	32.44***	28.38*	29.53
	< 18	1.7	2.5	0.4	0.1	2.4	0.8	1.6	0	0.9	0
	18–24	30.3	33.3	25.2	25.9	33.0	13.3	25.0	10.4	22.4	33.3
	25–29	32.4	31.5	34.0	33.8	42.4	34.0	43.0	13.0	32.7	13.3
	30–34	22.8	20.4	27.0	27.2	17.0	34.4	16.4	39.1	31.8	20.0
	≥ 35	12.8	12.3	13.5	13.0	5.2	17.4	14.1	37.4	12.1	33.3
Age moved to the UK (years)	< 4			8.3	8.9	1.4	3.3	28.9	0.9	6.5	0
	4–11			7.7	8.3	1.7	4.1	14.1	7.0	4.7	6.7
	12–17			7.6	7.8	7.9	7.1	9.4	3.5	2.8	20.0
	≥ 18			73.6	72.4	85.0	84.2	39.8	86.1	81.3	73.3
	Missing			2.8	2.6	3.8	1.2	7.8	2.6	4.7	0
Ethnicity	White British	39.5	61.3	2.0	0.1	6.3	4.6	37.5	0.9	1.9	0
	White other	2.7	0.3	6.8	0.1	85.1	1.7	24.2	0	1.9	0
	Asian	52.4	34.7	83.0	99.6	0.7	5.8	26.6	87.8	41.1	6.7
	Black	2.1	0.6	4.8	0	0	79.7	0	0	0.9	46.7
	Mixed – White & Asian	0.6	0.8	0.1	0.1	0	0	3.1	0	0	0
	Mixed – White& Black	1.0	1.5	0.1	0	0	0.8	1.6	0	1.9	0
	Other	1.7	0.8	3.2	0.2	8.0	7.5	7.0	11.3	52.3	46.7
Marital status	Married	65.6	51.7	89.4	95.9	52.4	60.6	60.9	88.7	93.5	73.3
	Cohabiting	17.8	25.5	4.4	0.2	35.4	14.5	23.5	6.1	3.7	6.7
	Single	16.4	22.5	6.0	3.8	11.5	24.9	25.6	4.3	2.8	20.0
	Missing	0.2	0.2	0.2	0.2	0.7	0	0	0.9	0	0
Employed/student		44.0	55.9	23.5	14.1	72.6	57.3	55.5	74.8	8.4	40.0
IMD^a^ quintile	5 (least deprived)	1.7	2.4	0.4	0.2	0.7	0.8	3.9	0	1.9	6.7
	4	2.9	4.2	0.8	0.6	1.0	1.2	0.8	2.6	0.9	0
	3	11.0	14.3	5.3	5.0	6.3	5.4	9.4	6.1	6.5	0
	2	18.0	19.5	15.6	14.6	23.3	10.0	22.7	27.8	15.9	20.0
	1 (most deprived)	66.4	59.7	78.0	79.7	68.8	82.6	63.3	63.5	74.8	73.3

*Note:* Level of significance: *p < 0.05, **p < 0.01, ***p < 0.001.

^a^ IMD: Index of Multiple Deprivation, is the official measure of relative deprivation for small, fixed areas/neighborhoods in England.

**TABLE 2:** Maternal health and gestational characteristics of the study population.

Variable	Category	Total population *n* = 11,361	UK born women *n* = 7183 (63.2%)	Migrant women *n* = 4178 (36.8%)	South Asia *n* = 3284 (28.9%)	Central Europe, Eastern Europe, and Central Asia *n* = 288 (2.5%)	Sub‐Saharan Africa *n* = 241 (2.1%)	High‐income *n* = 128 (1.1%)	Southeast Asia, East Asia, and Oceania *n* = 115 (1.0%)	North Africa and Middle East *n* = 107 (0.9%)	Latin America and Caribbean *n* = 15 (0.1%)
Booking BMI	Mean	26.04	26.45	25.33***	25.23***	23.89***	27.27	27.41	25.01*	26.20	26.71
	Underweight	4.0	3.2	5.3	6.1	4.5	2.1	0	3.5	0.9	0
	Healthy weight	41.9	40.9	43.5	43.1	58.3	30.3	39.1	52.2	39.3	40.0
	Overweight	26.7	26.4	27.2	27.7	20.5	28.6	26.6	26.1	29.9	20.0
	Obese	19.6	21.8	15.8	15.6	8.3	22.4	25.8	12.2	18.7	20.0
	Missing	7.9	7.7	8.2	7.5	8.3	16.6	8.6	6.1	11.2	20.0
Diabetes		8.4	6.3	12.0	13.1	5.6	6.6	8.6	18.3	4.7	13.3
Hypertensive disorders		6.3	6.5	6.3	5.8	6.6	6.6	9.4	10.4	2.8	0
Smoking during pregnancy		16.5	24.3	2.9	1.0	20.5	2.1	14.8	0.9	2.8	0
Parity	0	43.5	47.5	36.8	32.3	70.8	43.6	46.1	48.7	41.1	46.7
	1	27.7	28.3	26.6	26.0	21.2	35.3	28.1	31.3	32.7	26.7
	2 or more	28.8	24.2	36.6	41.7	8.0	21.2	25.8	20.0	26.2	26.7
Gestational age (weeks)	Mean	39.11	39.14	39.06*	39.04*	39.30	38.87*	39.24	38.84	39.57*	38.60
	< 37	6.0	6.3	5.5	5.1	6.6	9.1	6.3	6.1	1.9	13.3
	37–41	92.6	92.2	93.2	93.7	92.0	89.2	90.6	92.2	96.3	86.7
	≥ 42	1.4	1.5	1.3	1.2	1.4	1.7	3.1	1.7	1.9	0
Infant birthweight (g)	Mean	3225.21	3259.25	3166.70***	3129.40***	3372.46**	3236.10	3333.87	3222.38	3361.21	3027.33
	< 2500	8.0	7.8	8.4	8.9	6.9	8.3	4.7	5.2	2.8	20.0
	2500–3999	84.5	83.5	86.2	86.8	81.9	81.3	86.7	88.7	87.9	80.0
	≥ 4000	7.5	8.8	5.4	4.3	11.1	10.4	8.6	6.1	9.3	0
Size for gestational age	SGA	12.1	10.6	14.6	16.1	8.3	10.0	9.4	8.7	8.4	30.0
	AGA	73.9	73.8	74.1	74.5	69.4	73.0	71.1	80.0	73.8	80.0
	LGA	14.0	15.6	11.2	9.4	22.2	17.0	19.5	11.3	17.8	0

*Note:* Level of significance: *p < 0.05, **p < 0.01, ***p < 0.001.

C‐section births by population group including unadjusted OR is provided in Table 3. Migrants as a collective population were less likely to have a C‐section than UK born women (OR 0.88; 95% CI 0.80–0.97) and this was largely due to their significantly reduced likelihood of an elective C‐section (OR 0.82; 95% CI 0.70–0.94). This was also shown in the group encompassing migrants from Central Europe, Eastern Europe, and Central Asia; total C‐section OR 0.73 (95% CI 0.54–0.99), elective C‐section OR 0.39 (95% CI 0.21–0.73). Migrants from South Asia showed significantly lower incidence of C‐section (OR 0.80; 95% CI 0.72–0.89) due to both lower elective and emergency C‐sections, OR 0.75 (95% CI 0.64–0.88) and OR 0.87 (95% CI 0.77–0.98) respectively. Migrants from Sub‐Saharan Africa contrasted this showing a significantly higher incidence of C‐section (OR 1.74; 95% CI 1.33–2.29), due to higher rates for both elective (OR 1.71; 95% CI 1.17–2.49) and emergency C‐section (OR 1.52; 95% CI 1.11–2.10).

**TABLE 3:** Frequency and crude ORs for Caesarean section births by mother's region of origin.

Mother's origin by GBD region	Number of births	Total C‐section			Elective C‐section			Emergency C‐section		
		*n*	%	OR (95% CI)	*n*	%	OR (95% CI)	*n*	%	OR (95% CI)
Total population	11,361	2539	22.3		903	7.9		1637	14.4	
UK (ref)	7183	1663	23.2		610	8.5		1053	14.7	
All migrants	4178	876	21.0	0.88 (0.80–0.97)	293	7.0	0.82 (0.70–0.94)	584	14.0	0.95 (0.85–1.06)
South Asia	3284	639	19.5	0.80 (0.72–0.89)	213	6.5	0.75 (0.64–0.88)	427	13.0	0.87 (0.77–0.98)
Central Europe, Eastern Europe, and Central Asia	288	52	18.1	0.73 (0.54–0.99)	10	3.5	0.39 (0.21–0.73)	42	14.6	0.99 (0.71–1.39)
Sub‐Saharan Africa	241	83	34.4	1.74 (1.33–2.29)	33	13.7	1.71 (1.17–2.49)	50	20.7	1.52 (1.11–2.10)
High‐income	128	36	28.1	1.30 (0.88–1.92)	14	10.9	1.32 (0.76–2.31)	22	17.2	1.21 (0.76–1.92)
Southeast Asia, East Asia, and Oceania	115	35	30.4	1.45 (0.97–2.17)	12	10.4	1.26 (0.69–2.30)	23	20.0	1.46 (0.92–2.31)
North Africa and Middle East	107	25	23.4	1.01 (0.65–1.59)	11	10.3	1.24 (0.66–2.32)	14	13.1	0.88 (0.50–1.54)
Latin America and Caribbean	15	6	40.0	2.21 (0.79–6.23)	0	0		6	40.0	3.88 (1.38–10.93)

Abbreviation: GBD, global burden of disease.

Adjusted ORs are reported in Table 4. The impact of adjustment varies across population groups and for whether total, elective, or emergency C‐section is being examined. However, there are some populations of note. Women from South Asia continue to demonstrate a lower incidence of C‐section, except for Model 2 where the statistical significance of a lower total C‐section rate is lost. This may be a result of aOR now showing a higher rate of emergency C‐section although it does not reach the statistical significance level (aOR 1.03; 95% CI 0.90–1.18), as elective C‐section remains significantly lower (aOR 0.74; 95% CI 0.62–0.88). Women from “Central Europe, Eastern Europe, and Central Asia” have a significantly lower rate of total C‐section in Model 2 (aOR 0.72; 95% CI 0.52–0.99) resulting from much lower rates for elective and emergency C‐sections compared to crude ORs (aOR 0.45; 95% CI 0.24–0.87 and aOR 0.89; 95% CI 0.63–1.25, respectively). In contrast to these lower rates of C‐section, women from Sub‐Saharan Africa continue to demonstrate significantly higher rates for total C‐section (Model 1: aOR 1.52 (95% CI 1.15–2.01); Model 2: aOR 1.38 (95% CI 1.04–1.84)). However, whilst still showing higher rates than UK born women for elective and emergency C‐section, this difference only remains statistically significant with regards to Model 1, emergency C‐section (aOR 1.43 (95% CI 1.04–1.86)).

**TABLE 4:** Adjusted ORs for Caesarean births by mother's region of origin.

Mother's region of origin	Adjusted OR (95% CI)					
	Total C‐section		Elective C‐section		Emergency C‐section	
	Model 1	Model 2	Model 1	Model 2	Model 1	Model 2
All migrants	0.86 (0.78–0.94)	0.92 (0.83–1.03)	0.76 (0.66–0.89)	0.80 (0.67–0.93)	0.96 (0.86–1.07)	1.03 (0.91–1.17)
South Asia	0.79 (0.71–0.88)	0.89 (0.79–1.00)	0.70 (0.60–0.83)	0.74 (0.62–0.88)	0.88 (0.78–1.00)	1.03 (0.90–1.18)
Central Europe, Eastern Europe, and Central Asia	0.85 (0.63–1.16)	0.72 (0.52–0.99)	0.49 (0.26–0.94)	0.45 (0.24–0.87)	1.08 (0.78–1.51)	0.89 (0.63–1.25)
Sub‐Saharan Africa	1.52 (1.15–2.01)	1.38 (1.04–1.84)	1.40 (0.95–2.06)	1.45 (0.98–2.15)	1.43 (1.04–1.86)	1.23 (0.88–1.71)
High‐income	1.24 (0.83–1.83)	1.14 (0.76–1.72)	1.21 (0.68–2.15)	1.15 (0.64–2.07)	1.16 (0.74–1.97)	1.06 (0.66–1.73)
Southeast Asia, East Asia, and Oceania	1.23 (0.82–1.85)	0.94 (0.61–1.43)	0.88 (0.48–1.63)	0.85 (0.45–1.58)	1.42 (0.89–2.26)	1.03 (0.63–1.67)
North Africa and Middle East	0.95 (0.60–1.50)	1.01 (0.64–1.62)	1.15 (0.48–1.63)	1.24 (0.65–2.38)	0.86 (0.49–1.51)	0.88 (0.49–1.58)
Latin America and Caribbean	1.98 (0.69–5.70)	1.90 (0.65–5.58)			3.69 (1.31–10.46)	3.64 (1.25–10.62)

*Note:* Model 1: adjusted for maternal age and booking BMI. Model 2: adjusted for maternal age, booking BMI, Index of Multiple Deprivation (IMD) quintile, diabetes (pre‐existing and gestational), hypertensive disorders (pre‐existing and pregnancy related but excluding those which occurred in labor only), smoking during pregnancy, parity, gestational age, and infant birthweight.

## 4 Discussion

This study showed lower rates of C‐section amongst migrant populations collectively compared to UK born women. However, there were significant variations in the incidence of C‐section birth amongst different regional migrant groups with some demonstrating higher and others lower rates than UK born mothers. This signifies the importance of investigation according to specific identities such as country of origin or regional classifications rather than using collective “migrant” terminology which can be meaningless or misleading.

The observed variations in the total C‐section rates could be attributed to differences in the rates of either elective or emergency C‐sections alone, or a combination of differing rates for both, and this varied between regional migrant populations. Differences are largely maintained despite adjustments indicating that regional country of origin is a factor that influences the incidence of C‐section.

Caesarean section remains a vital procedure for both women and babies where it is medically indicated and can be a life‐saving procedure, though it is not without potential risks to both mother and baby. C‐section should therefore be reserved for those cases where it is truly indicated to minimize unnecessary risk. To address increasing global rates, it is essential to understand existing trends within and between countries which requires identifying populations with significantly differing rates, and undertaking work with such groups to understand health and social factors and decision‐making processes that result in these differences.

This analysis adds detail regarding the UK context in relation to migrants complementing findings from the few existing UK studies, allowing some direct comparison between specific migrant groups. Variation between different countries of origin and regional groupings has been reported for other host countries and findings from this study further add to this growing understanding of migrant women and incidence of C‐section [27, 28, 29, 30, 31, 32, 33, 34, 35, 36]. This is particularly important for migrant groups who consistently demonstrate the same pattern of difference in receiving countries when compared to women born within that country itself. Our data, like others [32, 33, 36, 37, 38], demonstrates that women from Sub‐Saharan African countries experience higher rates of C‐section. This particular trend amongst women from Sub‐Saharan African countries requires closer examination as many of the countries included in this region have the lowest national C‐section rates globally [3]. This could imply that the experience of migration and resettlement has a greater impact on women from this group. Further root cause analysis to explore contributing factors to such variations in whether cultural influences such as “Western acculturation” and coercion in adapting surgical procedures, or improved accessibility to maternity services play a role in women's choices of mode of birth are required. Another consideration with this regional group is the impact of practices surrounding female genital mutilation (FGM). FGM continues to be practiced in several countries, especially within Sub‐Saharan Africa [39]. Some studies with migrant mothers from these countries have reported a lack of awareness and familiarity amongst healthcare professionals (HCPs) regarding FGM practices that may contribute to a reluctance in managing labor and vaginal delivery. This in turn could be leading to higher rates of C‐section birth in these communities [40, 41].

For some migrant populations, it may be that a “healthy migrant” effect [42] whereby differences in lifestyle associated behaviors that contribute positively towards health are maintained or the migrating population consists of those that are healthier and more able to cope with the physical and/or sociological aspects of migration subsequently reduces the likelihood of C‐section within the migrating population. Whereas for others, the nature of the migratory journey and factors after arrival may itself contribute to additional risk. For example, migrants fleeing conflict or who have been trafficked are likely to have extra traumatic experiences impacting on both their physical and mental health. These groups are particularly vulnerable and more likely to be living in socio‐economic deprivation, with reduced accessibility to services due to factors such as language barriers, less awareness of service provision, mistrust or fear [43, 44, 45, 46].

However, reasons for such variations amongst populations are as yet mainly speculative with few known studies examining why this may be, either in relation to clinical factors that may predispose populations to certain conditions requiring intensive medical interventions, or the cultural factors that may be influencing a woman's decision making in relation to maternity care [10]. Of the studies found, the complexity of influencing factors is acknowledged with cultural factors from expectations, beliefs, practices, and prior experiences in the country of origin appearing to be a significant factor in how women view C‐section and therefore the decisions they make in their new country of residence [47, 48].

In terms of clinical practice and maternity service provision, the variation in rates of C‐section rates observed indicates that disparities between women from some migrant populations compared to UK counterparts exist. This requires further examination and intervention to reduce this. Interventions may include aspects such as improving awareness and accessibility of maternity provision that may differ markedly for a woman's country of origin, education of HCPs relating to cultural factors and issues such as FGM, and improved collaborative working between services to reduce disparities that may impact on health during pregnancy to facilitate better outcomes.

### 4.1 Strengths and Limitations

To our knowledge this is the first study in the UK investigating the C‐section mode of birth in a large diverse sample of women within the context of migration. This provides detailed subgroup comparisons based on regional classification of country of origin which could be helpful in informing future work and interventions to address widening gaps in maternal and neonatal health in the UK. It also provides a foundation for observing trends in C‐section rates from changing migration patterns, the impact of socio‐economic factors, and the impact of other demographic and health factors.

There are however limitations to the study. Firstly, the data utilized was collected between 2007 and 2011. During the time that has elapsed since data collection and our analysis, the overall migrant population has increased [21]. In addition to more usual economic reasons for migration, several distinctive factors affecting migration flow to the UK have occurred. These include new areas of political instability, war and conflict, the exit of the United Kingdom from the European Union, changes in immigration policy, and the global COVID‐19 pandemic. These scenarios impact on an individual's ability, opportunity, and/or necessity to leave their home country and migrate to another, as well as their health and wellbeing prior to, during, and following migration. It is recognized that in terms of country of birth, census data indicates the composition of migrant populations utilized in this analysis remains comparable to the current population [21]. However, the changing migratory circumstances of more recent populations could impact on the health of migrating women and subsequent birth outcomes which requires further exploration.

Secondly, the data set lacked information that would have been particularly useful for analysis. The most notable of these would be a record of previous C‐section, which is a well‐known risk factor for repeated C‐section. It would also have been beneficial to have a clear record of the category of C‐section alongside clinical indication and Robson classification to allow for a better understanding of contributing risk factors.

Finally, it is recognized that as well as the observed variation between the regional groupings utilized, the multiple countries within these will also exhibit variation within the regional grouping. Therefore, whilst our results provide an indication of the overall pattern from a global region, this may be different to an individual country within the regional group. Ideally, migrant populations should be analyzed at country level to better represent reality; however, this was not possible with the small numbers of women for many of the countries included in the data.

Overall, we envisage that our work has applicability to the rest of the UK, and thus provides a useful foundation for informing practice and future work.

## 5 Conclusion

Significant variations were observed in C‐section rates amongst migrant mothers and were specifically higher in women from Sub‐Saharan African countries compared to UK born mothers. Further work is required to inform the UK context with regards to Caesarean section birth and factors that influence this. Exploratory work should be conducted around the birth experience of migrant women and their experience of maternity services that could inform the interpretation of observed differences in C‐section rates. This could in turn inform the design and management of maternity provision to ensure a positive birth outcome and experience for migrant women.

## Funding

This analysis was carried out as part of a PhD programme of study supported by Sheffield Hallam University. Born in Bradford received funding from a Wellcome Trust infrastructure grant (WT101597MA), the National Institute for Health Research under its Yorkshire and Humber Applied Research Collaboration (NIHR200166, formerly Collaboration for Applied Health Research and Care, CLAHRC), and the NIHR Clinical Research Network which provided research delivery support for this study. The views expressed in this publication are those of the authors and not necessarily those of the National Institute for Health and Care Research or the Department of Health and Social Care.

## Conflicts of Interest

The authors declare no conflicts of interest.

## Supporting information


**Table S1:** Countries of origin of the study population by GBD region and super region.

## Data Availability

Analysis utilizes an existing dataset from the Born in Bradford cohort study.
