# Supplementary material for: Migration and Caesarean Section Birth in the United Kingdom: A Secondary Analysis of Born in Bradford Data
Source: Birth. 2026 Feb 4;53(3):566–76. doi: 10.1111/birt.70052 (PMC13471797; doi:10.1111/birt.70052)
Supplement: Supplementary file 1 [file BIRT-53-566-s001.docx]

Table S1. Countries of origin of the study population by GBD region and super region.

| **GBD^a^ super region** | **GBD^a^ region** | **Country of origin of migrant women included in analysis** |
| --- | --- | --- |
| South Asia | South Asia | Bangladesh, India, Pakistan |
| Central Europe, Eastern Europe, and Central Asia | Central Europe | Albania, Bosnia and Herzegovina, Bulgaria, Czech Republic, Kosovo, Poland, Romania, Serbia, Slovakia |
|  | Eastern Europe | Estonia, Latvia, Lithuania, Russia, Ukraine |
|  | Central Asia | Kazakhstan |
| Sub-Saharan Africa | Eastern Sub-Saharan Africa | Burundi, Eritrea, Ethiopia, Kenya, Malawi, Rwanda, Somalia, Sudan, Tanzania, Uganda, Zambia |
|  | Central Sub-Saharan Africa | Angola, Congo, Democratic Republic of Congo |
|  | Southern Sub-Saharan Africa | Botswana, South Africa, Swaziland (Eswatini), Zimbabwe |
|  | Western Sub-Saharan Africa | Benin, Cameroon, Gambia, Ghana, Guinea, Ivory Coast, Liberia, Nigeria, Senegal, Sierra Leone |
| High-income | Australasia | Australia, New Zealand |
|  | High-income Asia Pacific | Japan, Singapore |
|  | High-income North America | Canada, United States |
|  | Southern Latin America | None |
|  | Western Europe | Belgium, Cyprus, Demark, Finland, France, Germany, Gibraltar, Greece, Israel, Italy, Netherlands, Norway, Portugal, Republic of Ireland, Spain, Sweden, Switzerland |
| Southeast Asia, East Asia, and Oceania | Southeast Asia | Indonesia, Malaysia, Maldives, Mauritius, Philippines, Sri Lanka, Thailand, Vietnam |
|  | East Asia | China, Hong Kong, Taiwan |
|  | Oceania | None |
| North Africa and Middle East | North Africa and Middle East | Afghanistan, Algeria, Bahrain, Egypt, Iran, Iraq, Jordan, Kuwait, Libya, Morocco, Oman, Qatar, Saudi Arabia, Syria, Tunisia, Turkey, United Arab Emirates |
| Latin America and Caribbean | Andean Latin America | Peru |
|  | Central Latin America | Colombia, Mexico, Panama |
|  | Tropical Latin America | Brazil |
|  | Caribbean | Barbados, Dominica, Guyana, Jamaica, Trinidad |

a: Global Burden of Disease
